# Variational approach for Stokes flow through a two-dimensional non-uniform channel

**DOI:** 10.1038/s41598-024-66500-4

**Published:** 2024-07-08

**Authors:** Abhishek Banerjee, Alexander Oron, Yehuda Agnon

**Affiliations:** 1 Department of Mathematics, SRM Institute of Science and Technology Kattankulathur, Chennai, 603203 India; 2 Faculty of Mechanical Engineering, Technion - Israel Institute of Technology, Haifa, 3200003 Israel; 3 Faculty of Civil and Environmental Engineering, Technion - Israel Institute of Technology, Haifa, 3200003 Israel

**Keywords:** Stokes flow, Variational calculus, Euler-Lagrange equation, Finite volume method, Engineering, Mathematics and computing

## Abstract

A variational approach is proposed to study the Stokes flow in a two-dimensional non-uniform channel. By using the stationarity of the Lagrangian, the Euler-Lagrange equations are established which leads to a simple set of ordinary differential equations to provide an estimate for the average pressure drop explicitly in terms of the channel shape function. The results for the pressure drop show an excellent agreement with the second-order extended lubrication theory. A higher-order formulation further improves the accuracy of the results for the pressure drop along the channel.

## Introduction

Stokes flow in confined geometries is encountered in microfluidics^1^, lab-on-chip technologies^2^, medical sciences^3,4^, design of innovative micro-devices to carry drugs^5,6^, biological systems^7,8^ and many other applications.

A number of analytical methods exist for solving two-dimensional Stokes flows in different channel geometries. Davis^9^ used Fourier transforms to provide answers to a number of point singularity problems in a channel. Problems involving mixed boundary conditions can be analysed using the Wiener-Hopf technique, which is based on Fourier transforms and the factorization of functions into upper and lower analytic ones. The flow in a channel split by a semi-infinite wall is a traditional problem. Analysis of the symmetric channel divider reduces to a scalar Wiener-Hopf problem, which was initially solved by Foote & Buchwald^10^ and Buchwald & Doran^11^. Jeong^12^ also used the Wiener-Hopf technique to evaluate the flow around the semi-infinite wall in a symmetric channel divider. In an asymmetric channel divider, Stokes flow was taken into consideration by Abrahams et al.^13^. Using Pad$\acute{e}$ approximations, the boundary value problem was reduced to a matrix Wiener-Hopf problem. Kim & Chung^14^ used a three-part Wiener-Hopf formulation to analyse the flow in a channel with a finite plate parallel to the walls. Series expansions in rectangular regions are another method used to tackle difficulties in channel geometries. To investigate the flow in a channel with a contraction, Phillips^15^ employed the technique of matching eigenfunction expansions utilising Papkovich-Fadle functions. Motivated by biological applications, Setchi et al.^16^ solved for a range of low Reynolds-number flows in a channel through a shunt using Papkovich-Fadle eigenfunctions connected to semi-strip geometries. Boundary integral methods can be utilised to examine the resulting flows for more complex geometries^17,18^.

Another class of solution methods to solve Stokes flow problems in confined geometries is Classical Lubrication Approximation (CLA). The CLA, as it allows one to stay away from carrying out a full-scale fluid calculation, is used in many fields such as film lubricant^19^, hydraulic fracture mechanics^20,21^, dykes and sills in volcanism^22^, or flows in biological systems such as blood cell transport in narrow capillaries^23^. When the curvature of the channel is no longer negligible, its validity as that of the related Reynolds equation must be questioned^24,25^. Because the channel is thinner than the other dimensions, asymptotic approaches are a good fit for doing this in a methodical manner. Applying an Extended Lubrication Approximation (ELA) to channels with a single wavy wall^26^ and two symmetric wavy walls^27^ has been a practice since the 1980s. In these papers, power series of the stream function have been obtained for low Reynolds-number flows and the results have been applied to studies of hydraulic fracture mechanics, e.g., Sisavath et al.^28^ and references herein. The work of Fabricius et al.^29^ on roughness is also mentioned, where the macroscopic effective behaviour is extracted by combining homogenization with asymptotic analysis. Recently, Tavakol et al.^30^ introduced an approximation to higher-order terms, or extended lubrication theory (ELT), to resolve shortcomings in the classic lubrication theory. The focus of their analysis was low Reynolds number flows in channel geometries whose boundaries were represented by continuous, differentiable, or piecewise differentiable form functions. Their analytical result is supported by experimental and computational data, which further highlights the upgraded model’s interest in precisely capturing the velocity profiles and pressure drop along a channel. Luca & Llewellyn Smith^31^ solved Stokes-flow equations in a two dimensional channel with a linear expansion using the Unified Transformation Method (UTM) by splitting the domain into three convex sub-domains, providing quasi-analytical solutions. The results obtained using the UTM for the pressure drop were compared to those obtained using the ELT adapting the analysis of Tavakol et al.^30^. Hinojosa et al.^32^ recently presented an extended lubrication approximation for arbitrary shapes while the work of Tavakol et al.^30^ was restricted to asymmetric channels. Few more studies on lubrication approximation for Newtonian and viscoelastic rheology were reported recently by Christov et al.^33^, Dewangan & Datta^34^, Boyko & Stone^35^ and in the references there.

Variational principles often play an important role in basic physics to provide a deeper understanding of natural laws. In research based on variational principles, there are two groups of approaches: the first one finds the minimal entropy production for dissipative systems, while the second one is created from the minimum action integral in classical mechanics of energy-conserving systems. Fermat’s principle, which is analogous to Snell’s rule of light refraction and was put forth in the 17th century to anticipate the path of light, is where the minimum action integral got its start. Hamilton and other scientists later extended Maupertuis’ concept of the minimum action integral in mechanics, which was based on this theory. The second set of variational principles originates from the work of Prigogine and his associates^36^, wherein systems with thermal nonequilibrium are crucially dependent on the condition of minimum entropy production. A variational principle is applied to the problem of instability in the context of thermal convection^37–39^. The equilibrium between the work performed by the buoyancy force and the viscous dissipation in the convective flow is the critical condition of the Benard convection, and the critical Rayleigh number is determined by the stationary value of the ratio of these quantities^37^. Takaki^40^ used the variational principle, which necessitates the stationarity of the difference between the work performed by the pressure force and the entropy production, to construct the Stokes equations and the continuity equation. Wang et al.^41^ established the form of the Lagrangian function using the variational principle. Other recent use of variational approach can be found in the works of Xu et al.^42^ and Zhou & Doi^43^.

**Figure 1:**
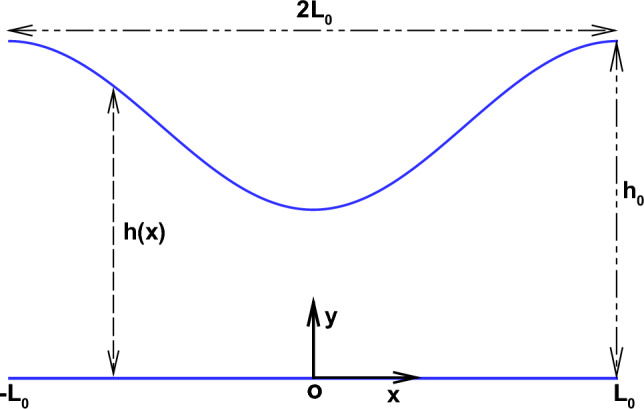
Sketch of the problem.

In this paper, we focus on the Stokes flow in a two-dimensional non-uniform channel, as shown schematically in Fig. 1. The problem is analyzed by constructing a functional, named Lagrangian for Stokes flow using the variational principle proposed by Takaki^40^ and was implemented by Wang et al.^41^. Considering the stationarity of the Lagrangian with respect to the set of unknown variables, the Euler-Lagrange equation which estimates the average pressure drop between the two ends of the non-unform channel is formulated. We compare our results with those obtained from the second-order ELT presented by Tavakol et al.^30^. We also perform finite volume numerical simulations based on the solution of Stokes equations to validate our results.

This paper is organized as follows. In the section ’Formulation of the problem and mathematical model’, the problem of Stokes flow in a two-dimensional non-uniform channel is formulated using the variational approach. Section ’Numerical simulations’ describes the details of the numerical method used here. The results obtained in terms of variation of the pressure drop for a constricting channel are presented in the section ’Variation of pressure drop for a constricting channel’. The results for the pressure drop variation in an expanding channel are described in section ’Variation of the pressure drop for an expanding channel’. Section ’Estimate of the improvement of the solutions’ presents the estimation and prediction of the improved results obtained in terms of the pressure drop using the velocity profile proposed by Hinojosa et al.^32^. The salient conclusions of the paper are summarised in section ’Conclusions’.

## Formulation of the problem and mathematical model

We consider a steady, two-dimensional pressure-driven flow of an incompressible Newtonian fluid in a channel of length $2 L_0$ bounded by a planar horizontal wall on one side and a corrugated wall on the other. The material properties of the liquid are density $\rho$, dynamic viscosity $\mu$ and the kinematic viscosity $\nu =\mu /\rho$. The x-axis of the chosen reference frame coincides with the planar wall and the y-axis is normal to the x-axis and points into the fluid. The location of the channel walls are given by $y=0$ and $y=h(x)=h_0 H(X)$, where $h_0$ is a characteristic channel height and *H*(*X*) is the dimensionless channel height.

The wall shape function *h*(*x*) is assumed to be at least $C^2$ continuous in the entire stretch of the channel. We also assume that the Reynolds number $R=Q/\nu$ based on the volumetric flow rate through the channel *Q* is small, so the flow is governed by Stokes equations

1 $$\begin{aligned} \mu {\nabla }^2 \varvec{u}=\nabla p, ~~~~~~~ \nabla \cdot \varvec{u}=0, \end{aligned}$$

where $\varvec{u}=(u,v)$ is the velocity field, *p* is the pressure and $\mu$ is the fluid dynamic viscosity.

The Classical Lubrication Approximation (CLA) yields^30,32^

2 $$\begin{aligned} u= & {} \left( \frac{6 Q}{h(x)}\right) \left( \frac{y}{h(x)}\right) \left( 1-\frac{y}{h(x)}\right) , \end{aligned}$$

3 $$\begin{aligned} v= & {} 0, \end{aligned}$$

4 $$\begin{aligned} ~~\frac{\partial {p}}{\partial {y}}= & {} 0, \end{aligned}$$

which can be simplified as

5 $$\begin{aligned} u= & {} 6 \hat{u}(x) \left( \frac{y}{h(x)}\right) \left( 1-\frac{y}{h(x)}\right) , \end{aligned}$$

6 $$\begin{aligned} v= & {} 0, \end{aligned}$$

7 $$\begin{aligned} p= & {} g(x), \end{aligned}$$

where $\hat{u}(x)=Q/h(x)$ and *g*(*x*) are functions of *x* which are to be determined.

Following Takaki^40^ and Wang et al.^41^ we construct the Lagrangian for Stokes flow in the corrugated domain as

8 $$\begin{aligned} \mathscr {L}= & {} \int _{0}^{h(x)} \left\{ \mu {[\nabla \cdot \varvec{u}]}^2 +\frac{1}{2}\mu \left( \frac{\partial {u}}{\partial {y}}+\frac{\partial {v}}{\partial {x}} \right) ^2 -p \nabla \cdot \varvec{u} \right\} dy \nonumber \\= & {} \int _{0}^{h(x)} \left\{ \mu {[\nabla \cdot \varvec{u}]}^2 \right\} dy + \int _{0}^{h(x)} \left\{ \frac{1}{2}\mu \left( \frac{\partial {u}}{\partial {y}}+\frac{\partial {v}}{\partial {x}} \right) ^2 \right\} dy -\int _{0}^{h(x)} \left\{ p \nabla \cdot \varvec{u} \right\} dy \nonumber \\\equiv & {} \mathscr {L}_1 + \mathscr {L}_2 - \mathscr {L}_3. \end{aligned}$$

Using the expressions (5)–(7) for the CLA of Stokes flow, we obtain

9 $$\begin{aligned} \mathscr {L}_1= & {} \int _{0}^{h(x)} \left\{ \mu {[\nabla \cdot \varvec{u}]}^2 \right\} dy \nonumber \\= & {} \frac{24 \mu }{5} \frac{\hat{u}(x)^2 h^{'}(x)^2}{h(x)} +\frac{6 \mu }{5} \hat{u}(x) \hat{u}^{'}(x)h^{'}(x) + \frac{6 \mu }{5} \hat{u}^{'}(x)^2 h(x), \end{aligned}$$

10 $$\begin{aligned} \mathscr {L}_2= & {} \int _{0}^{h(x)} \left\{ \frac{1}{2}\mu \left( \frac{\partial {u}}{\partial {y}}+\frac{\partial {v}}{\partial {x}} \right) ^2 \right\} dy \nonumber \\= & {} 6 \mu \frac{\hat{u}(x)^2}{h(x),} \end{aligned}$$

11 $$\begin{aligned} \mathscr {L}_3= & {} \int _{0}^{h(x)} \left\{ p \nabla \cdot \varvec{u} \right\} dy \nonumber \\= & {} g(x) \hat{u}(x)h^{'}(x) +g(x) \hat{u}^{'}(x)h(x). \end{aligned}$$

Combining the above integrals we obtain the Lagrangian $\mathscr {L}$ as a functional of $\hat{u}$ and *g* in the form

12 $$\begin{aligned} \mathscr {L} \equiv \mathscr {L} \left[ \hat{u}(x),g(x)\right]= & {} \frac{24 \mu }{5} \frac{\hat{u}(x)^2 h^{'}(x)^2}{h(x)} +\frac{6 \mu }{5} \hat{u}(x) \hat{u}^{'}(x)h^{'}(x) + \frac{6 \mu }{5} \hat{u}^{'}(x)^2 h^{'}(x)\nonumber \\{} & {} +6 \mu \frac{\hat{u}(x)^2}{h(x)} - g(x) \hat{u}(x)h^{'}(x) - g(x) \hat{u}^{'}(x)h(x). \end{aligned}$$

Using the methods of calculus of variations we obtain a set of two Euler-Lagrange equations as

13 $$\begin{aligned}{} & {} \frac{\partial }{\partial {g}} \mathscr {L} - \frac{d}{d x} \left( \frac{\partial {\mathscr {L}}}{\partial {g^\prime }} \right) =0, \end{aligned}$$

14 $$\begin{aligned}{} & {} \frac{\partial }{\partial {\hat{u}}} \mathscr {L} - \frac{d}{d x} \left( \frac{\partial {\mathscr {L}}}{\partial {{\hat{u}}^{\prime }} } \right) =0, \end{aligned}$$

which reduce to

15 $$\begin{aligned}{} & {} \hat{u}(x)h^{'}(x) + \hat{u}^{'}(x)h(x) =0, \end{aligned}$$

16 $$\begin{aligned}{} & {} g^{'}(x) h(x)+ 12 \mu \frac{\hat{u}(x)}{h(x)}+ \frac{48 \mu }{5} \frac{\hat{u}(x) h^{'}(x)^2}{h(x)}- \frac{12 \mu }{5}\hat{u}^{'}(x) h^{'}(x) - \frac{6 \mu }{5}\hat{u}(x) h^{''}(x)-\frac{12 \mu }{5}\hat{u}^{''}(x) h(x) =0, \end{aligned}$$

respectively. The first Euler-Lagrange Eq. (15) results in

17 $$\begin{aligned} \frac{d}{d x} \left( \hat{u}(x) h(x)\right) =0, \end{aligned}$$

which is automatically satisfied since $\hat{u}(x) h(x)=Q$ is a constant flow rate *Q* along the channel, and therefore

18 $$\begin{aligned} \hat{u}^{\prime }(x)=- \frac{Q}{h(x)^2} h^{\prime }(x) \mathrm{~{and}~} \hat{u}^{\prime \prime }(x)=\frac{Q}{h(x)^3} \left\{ 2 h^{\prime }(x)^2 -h(x)h^{\prime \prime }(x) \right\} . \end{aligned}$$

Substituting Eqs. (17) and (18) into the second Euler-Lagrange Eq. (16) we obtain

19 $$\begin{aligned} g^{\prime }(x)= -\frac{6 \mu Q}{5 h(x)^3} \left[ 6 h^{\prime }(x)^2+h(x)h^{\prime \prime }(x)+10 \right] . \end{aligned}$$

Hence, it is possible to obtain an expression for the average-pressure gradient $\bar{p}^{\prime }(x)$ along the channel

20 $$\begin{aligned} {\bar{p}^{\prime }(x)} = \frac{\partial }{\partial {x}} \left\{ \frac{1}{h(x)}\int _{0}^{h(x)} p ~ dy \right\} \end{aligned}$$

as

21 $$\begin{aligned} {\bar{p}^{\prime }(x)} \equiv \frac{\partial }{\partial {x}} \left\{ \frac{1}{h(x)} \int _{0}^{h(x)} g(x) dy \right\} = g^\prime (x) = -\frac{6 \mu Q}{5 h(x)^3} \left[ 6 h^{\prime }(x)^2+h(x)h^{\prime \prime }(x)+10 \right] . \end{aligned}$$

In order to obtain the dimensionless average pressure gradient, the following dimensionless variables are introduced

22 $$\begin{aligned} X=\frac{x}{L_0}, Y=\frac{y}{h_0}, U=\frac{u}{Q/h_0}, V=\frac{v}{Q/L_0}, \bar{P}^{\prime }=\frac{\bar{p}^{\prime }}{\mu Q L_0/h_0^3}. \end{aligned}$$

In terms of these non-dimensional variables, we obtain the dimensionless pressure gradient as

23 $$\begin{aligned} {\bar{P}^{\prime }(X)}= -\frac{6}{5 H(X)^3} \left[ 10+ {\delta }^2 H(X) H^{\prime \prime }(X)+6 {\delta }^2 H^{\prime }(X)^2 \right] \end{aligned}$$

where

24 $$\begin{aligned} \delta =h_0/L_0 \end{aligned}$$

is the aspect ratio of the channel. Thus, the pressure drop $\Delta \bar{P}$ can be determined as

25 $$\begin{aligned} \Delta \bar{P} = \int _{-1}^{1} {\bar{P}^{\prime }(X)} dX =-\frac{6}{5} \int _{-1}^{1} \frac{1}{H(X)^3} \left[ 10+ {\delta }^2 H(X) H^{\prime \prime }(X)+6 {\delta }^2 H^{\prime }(X)^2 \right] dX.~~~~~~~ \end{aligned}$$

## Numerical simulations

We compare our results for the pressure drop obtained analytically with the corresponding results obtained using direct numerical simulations. To do this, we solve numerically the full time-dependent Navier-Stokes equations for a two-dimensional incompressible flow in a channel with prescribed boundary conditions. As a result of this solution starting with an arbitrary chosen initial condition, we look for a steady solution of the system.

The continuity and Navier-Stokes equations (in the Stokes theory limit for the exceedingly small Reynolds number) are brought into dimensionless form using the scaling and dimensionless parameters introduced in Eqs. (22) and (24) to obtain

26 $$\begin{aligned} \frac{\partial U}{\partial X} + \frac{\partial V}{\partial Y}= & {} 0, \end{aligned}$$

27 $$\begin{aligned} {\delta }^2 \frac{{\partial }^2 U}{\partial {X}^2} + \frac{{\partial }^2 U}{\partial {Y}^2} -\frac{\partial P}{\partial X}= & {} 0, \end{aligned}$$

28 $$\begin{aligned} {\delta }^4 \frac{{\partial }^2 V}{\partial {X}^2} + {\delta }^2 \frac{{\partial }^2 V}{\partial {Y}^2} - \frac{\partial P}{\partial Y}= & {} 0. \end{aligned}$$

In order to convert the corrugated geometry of the channel shape into a conventional rectangular one a mapping^44,45^

29 $$\begin{aligned} \xi =X, ~~~~ \eta =\frac{Y}{H(X)} \end{aligned}$$

is applied to Eqs. (26)–(28).

Under the mapping (29), Eqs. (26-28) are recast as

30 $$\begin{aligned}{} & {} \frac{\partial U}{\partial \xi }- \frac{\eta H^{\prime }}{H} \frac{\partial U}{\partial \eta }+\frac{1}{H} \frac{\partial V}{\partial \eta }=0, \end{aligned}$$

31 $$\begin{aligned}{} & {} \quad {\delta }^2 \frac{{\partial }^2 U}{\partial {\xi }^2}-2{\delta }^2 \frac{\eta H^{\prime }}{H} \frac{{\partial }^2 U}{\partial \xi \partial \eta }+\frac{1}{H^2} \left( 1+{\delta }^2 {\eta }^2 H~'~^2 \right) \frac{{\partial }^2 U}{\partial {\eta }^2}\nonumber \\{} & {} \qquad +{\delta }^2 \eta \left( \frac{2}{H^2} H~'~ ^2 -\frac{1}{H} H^{\prime \prime } \right) \frac{\partial U}{\partial \eta } - \frac{\partial P}{\partial \xi } + \frac{\eta H^{\prime }}{H} \frac{\partial P}{\partial \eta }=0, \end{aligned}$$

32 $$\begin{aligned}{} & {} \quad {\delta }^4 \frac{{\partial }^2 V}{\partial {\xi }^2}-2{\delta }^4 \frac{\eta H^{\prime }}{H} \frac{{\partial }^2 V}{\partial \xi \partial \eta }+\frac{1}{H^2} \left( 1+{\delta }^4 {\eta }^2 H~'~^2 \right) \frac{{\partial }^2 V}{\partial {\eta }^2}\nonumber \\{} & {} \qquad +{\delta }^4 \eta \left( \frac{2}{H^2} H~'~^2 -\frac{1}{H} H^{\prime \prime } \right) \frac{\partial V}{\partial \eta }-\frac{1}{H} \frac{\partial P}{\partial \eta }=0. \end{aligned}$$

In our direct numerical simulations, a staggered-grid-based finite volume method (FVM) is employed to solve the set of Eqs. (30)–(32). By progressing the flow field variables via a series of lesser time steps of size 0.001, a time-marching numerical approach is obtained. It is found that the flow field achieves a steady state independent of the stipulated initial conditions after a brief transient for the range of parameter values studied here. A cyclic pressure correction approach, the SIMPLE algorithm, is used to solve the discretized Eqs. (30)–(32)^44–46^. The discretized continuity equation is transformed into a Poisson equation for the pressure correction, which is then solved iteratively using the SOR (Successive over relaxation) scheme until the desired accuracy is achieved. This creates the pressure link between the momentum and continuity equations. The convergence criterion is defined as $\max _{i,j} \mid \Theta _{i,j}^{(n+1)}-\Theta _{i,j}^{(n)}\mid <\Delta$, where $\Theta =(U, V, P)$, $\Delta = 10^{-6}$, *n* denotes the iteration number, and *i*, *j* stand for the number of the node of the computational grid. The numerical simulations are performed in the non-dimensional transformed domain $\left( \eta \in [-1,1] \right) \times \left( \xi \in [0,1]\right)$ with the non-uniform spacing with denser grid points in the wall vicinity to capture the gradients of the velocity components. The typical grid spacing used in our simulations in the $\eta$ direction is varied between 0.005 and 0.01, whereas the grid spacing in the $\xi$ direction is uniform in the range between 0.01 and 0.02. The dimensionless channel height is considered $H_{0}=1$. Additionally to a no-slip, no-penetration conditions on the walls, the boundary conditions are defined using the Poiseuille velocity profile at the inlet, and a vanishing outlet gauge pressure^30^. Thus, the boundary conditions in the transformed region $[-1,1] \times [0,1]$ takes the form

33 $$\begin{aligned} U(-1,\eta )=6 \eta (1-\eta ), ~ V(-1,\eta )=0, ~U(\xi ,0)=U(\xi ,1)=0, ~V(\xi ,0)=V(\xi ,1)=0, ~P(1,\eta )=0. \end{aligned}$$

**Figure 2:**
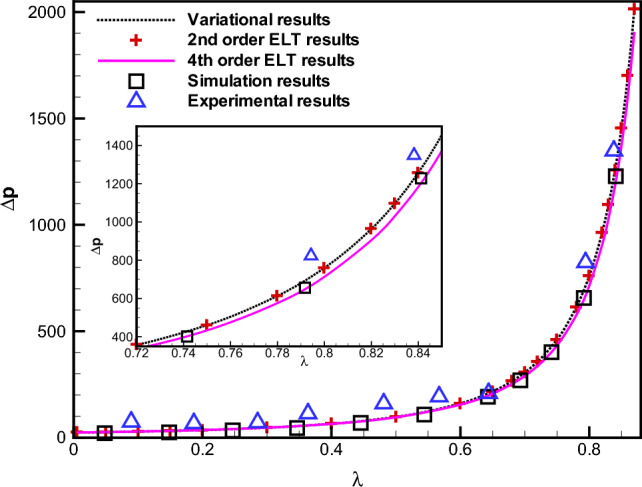
Comparison between the results for the dimensionless pressure drop obtained for $\delta =1$ using our variational approach based on the parabolic velocity profile (the dotted curve), our numerical finite-volume simulations (squares $\square$), and the second- ($+$) and fourth-order ELT (the solid curve) derived by Tavakol et al.^30^ for a constricting channel ($\lambda > 0$). The triangular symbols $\Delta$ represent the experimental results obtained by Tavakol et al.^30^.

**Figure 3:**
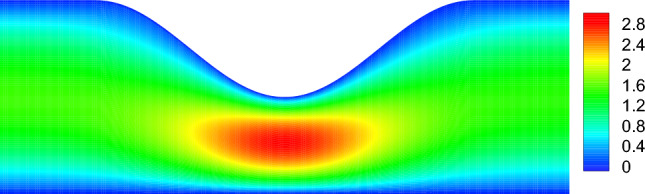
Velocity magnitude obtained using finite-volume simulations for $\lambda =0.5$ and $\delta =1$ for a constricting channel.

**Figure 4:**
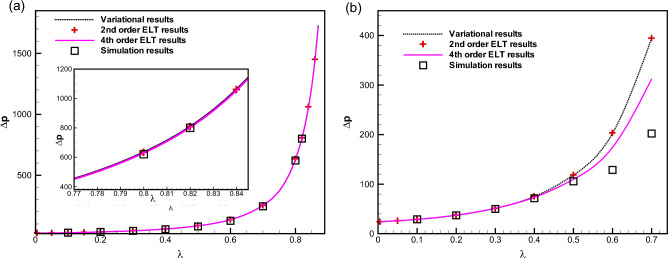
Comparison between the results for the dimensionless pressure drop obtained using our variational approach (the dotted curve), our numerical simulations (squares ($\square$), and the second- ($+$) and fourth-order (the solid curve) lubrication approximations for a constricting channel ($\lambda > 0$) derived by Tavakol et al.^30^. (**a**) $\delta =0.5$; (**b**) $\delta =1.5$.

## Results and discussion

In this section, we present our analytical results for the pressure drop based on the variational formulation derived in Section Formulation of the problem and mathematical model, for a non-uniform channel. We also aim to compare our results with those of the CLT (Classical Lubrication Theory) and a higher-order ELT (Extended Lubrication Theory) developed by Tavakol et al.^30^. We also validate the predictions of our variational model by performing 2D numerical simulations using the finite-volume approach described in Section Numerical simulations.

To validate and compare our results derived by the variational approach, we choose the same example considered by Tavakol et al.^30^ where the normalized wall shape function is given by

34 $$\begin{aligned} H(X)=1-\frac{\lambda }{2} \left( 1+\cos (\pi X) \right) ~~~~~~ \left( 0 \le \mid \lambda \mid < 1 \right) , \end{aligned}$$

where $\lambda$ represents the dimensionless amplitude of the channel height variation; $\lambda > 0$ and $\lambda < 0$ stand for the cases of constricting and expanding channel shapes, respectively. For the purpose of our fully numerical solution only we extend the channel by $H(X)=1$ for $\mid X \mid >1$.

For a fixed value of the flow rate *Q*, the dimensionless pressure drop between $X=-1$ and $X=1$ in the case of the shape function (34) is obtained by Tavakol et al.^30^

35 $$\begin{aligned} \Delta {P} = \Delta {P_0}+ {\delta }^2 \Delta {P_2}+ {\delta }^4 \Delta {P_4}, \end{aligned}$$

where $\Delta {P_0}$, $\Delta {P_2}$ and $\Delta {P_4}$ represent the pressure drop at leading order in $\delta$, and the second- and fourth-order lubrication approximation, respectively. The expressions for $\Delta {P_0}$, $\Delta {P_2}$ and $\Delta {P_4}$ are explicitly given in^30^

36 $$\begin{aligned} \Delta {P_0} = \frac{3(3 {\lambda }^2 -8 \lambda +8)}{(1- \lambda )^{5/2}}, \end{aligned}$$

37 $$\begin{aligned} \Delta {P_2} = \frac{12(\pi \lambda )^2}{5(1- \lambda )^{3/2}}, \end{aligned}$$

38 $$\begin{aligned} \Delta {P_4} = \frac{8 {\pi }^2 (428(-1+ \sqrt{1- \lambda }) -214(-2+ \sqrt{1- \lambda })\lambda -53 {\lambda }^2 )}{175 \sqrt{1- \lambda }}. \end{aligned}$$

### Variation of pressure drop for a constricting channel ($\lambda > 0$)

In this section, we explore the effect of the dimensionless amplitude $\lambda$ and the aspect ratio $\delta$ on the pressure drop in the case of the channel with a concave shape. We compare the values of the pressure drop derived using the variational approach with those obtained for the extended lubrication theory (ELT) of various orders^30^ and our simulation results.

First, in Fig. 2 we present the non-dimensional pressure drop $\Delta {P}=\Delta {p}/ (\mu Q L_0/h_0^3)$ between the two ends of the channel as a function of $\lambda$ with a fixed $\delta =1$. Our variational results along with those for various-order ELT together with our simulation results are all presented in Fig. 2. It is evident from this Fig. 2 that the pressure drop increases rapidly with an increase in $\lambda$, as expected, since the flow rate is constant through any cross section along the channel. The most important observation from Fig. 2 is that our results for the pressure drop derived from the variational approach agree very well with the second-order ELT for a wide range of $\lambda$ with $\delta =1$. However, it is also evident from Fig. 2 that the experimental results^30^ are in a better agreement with the second-order ELT (alternatively, with our variational results) rather than with those of the fourth-order ELT. A sample of our simulation results for the velocity magnitude with $\lambda =0.5$ and $\delta =1$ are presented in Fig. 3.

In Figs. 4a and b, we compare between our analytical results, the results of various- order ELT solutions and those of our numerical simulations. It is evident from Fig. 4a that when $\delta$ is small, e.g. $\delta =0.5$, the difference between the second- and fourth-order ELT results is negligible and they fit our results arising from our both variational and numerical simulation results very accurately. For higher values of $\delta$, say for $\delta =1.5$, one can observe that the results derived from the variational approach agree with those of the second-order ELT. It is important to mention that the non-dimensional pressure drop derived from the variational approach and various-order ELT deviate from the simulation results above a certain value of $\lambda$ (say, $\lambda =0.5$).

**Figure 5:**
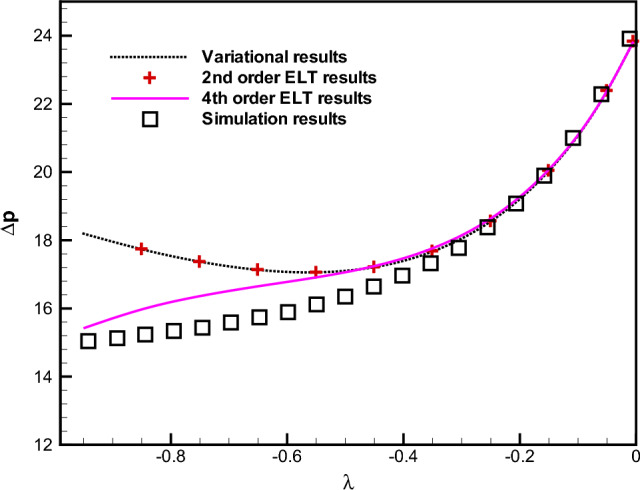
Comparison between the dimensionless pressure drop for $\delta =1$ as obtained from the variational approach based on the parabolic profile (the dotted curve), finite-volume simulation results (squares $\square$), and the second- ($+$) and fourth-order (the solid line) lubrication approximations obtained by Tavakol et al.^30^ for an expanding channel.

**Figure 6:**
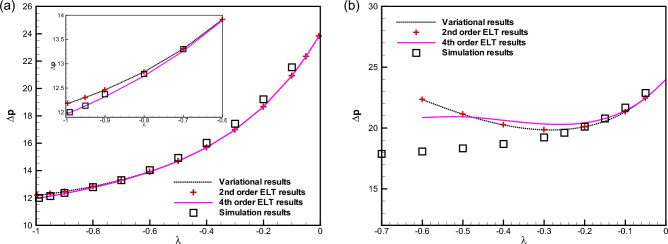
Variation of the dimensionless pressure drop with $\lambda$ as obtained using the variational approach based on the parabolic profile (the dashed curve), our finite-volume simulations ($\square$), and second- ($+$) and fourth-order (the solid curve) lubrication approximations both derived by Tavakol et al.^30^ for an expanding channel ($\lambda < 0$). Left panel: $\delta =0.5$; Right panel: $\delta =1.5$.

**Figure 7:**
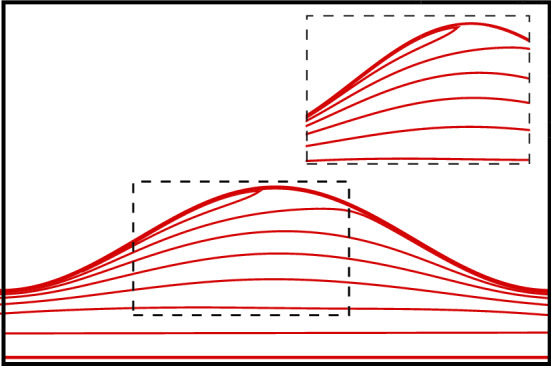
Streamlines map obtained using finite-volume simulations for $\lambda =-0.60$ and $\delta =1$. The inset shows a magnifying view of the streamlines near the reverse flow region.

### Variation of the pressure drop for an expanding channel ($\lambda < 0$)

This subsection is devoted to discussion of our results based on the variational formulation and to their comparison with various-order ELTs^30^ and with our numerical results for an expanding channel of a convex shape. Such comparison is depicted in Fig. 5 for a channel defined with a convex shape function ($\lambda < 0$) for $\delta =1$. The convexity of the shape alters the characteristics of the $\Delta p - \lambda$ dependence, as shown in Fig. 5. As $\lambda$ increases, the pressure drop between the two ends reduces to conserve the flow rate in an arbitrary cross section of the channel. It is evident from Fig. 5 that similar to the results obtained for contracting geometries discussed above, our variational results for the dimensionless pressure drop agree very well with the second-order ELT in case of an expanding channel for a wide range of $\lambda$ with $\delta =1$. However, for a channel with a sharp convexity, the results obtained from the fourth-order ELT show a better agreement with our numerical simulations.

Figure 6 depicts the non-dimensional pressure drop $\Delta p$ between the two ends of an expanding channel as a function of $\lambda$ for $\delta =0.5$ and $\delta =1.5$ in its left and right panels, respectively. It is observed in the left panel of Fig. 6 that for a small value of $\delta$, e.g., $\delta =0.5$, the differences between the results of our numerical simulations, and those of the second- and fourth-order ELT is very small and they agree with our variational results very well (within $4 \%$ error). For a higher value of $\delta$, e.g., $\delta =1.5$, the results are presented in the right panel of Fig. 6. The comparison reveals that our variational results for the pressure drop agree very well with the second-order ELT for a broad range of $\lambda$, even for $\delta > 1$. However, our simulation results agree with our variational results (alternatively, with the second-order ELT) up to a small value of $\lambda$, $0>\lambda >-0.3$ (within $5 \%$ accuracy), and the discrepancy begins to increase rapidly with an increase in the channel convexity. It is also observed from the right panel of Fig. 6 that in the case of an expanding channel with $\delta =1.5$, the fourth-order ELT can not provide a better estimate than the second-order ELT in the range of moderate $\lambda$.

Finally, one should bear in mind that the case of an expanding channel represents, in general, a more complex setting than that of a constricting one. The possibility of a flow separation may invalidate the variational approach based on a parabolic velocity profile. Figure 7 shows the evidence of flow separation near the wall crest of the channel for an expanding channel with $\lambda =-0.60$ and $\delta =1$, as obtained by our fully numerical solution of the flow. Flow reversal magnified in the inset to Fig. 7 for a better view causes a significant difference between the fully numerical results and our results based on the variational approach for a higher value of $\mid \lambda \mid$. It is worth mentioning that for a low aspect ratio of the channel, e.g., $\delta =0.5$, flow separation does not take place, and thus our variational results are expected to show a good agreement with fully numerical results, as seen in Fig. 6a. However, in the case of a higher aspect ratio, e.g., $\delta =1.5$, flow separation becomes important to make a significant difference between the variational and numerical results, as observed in Fig. 6b.

### Estimate of the improvement of the solutions

In the previous sections, we have observed that the analytical results for the pressure drop obtained using the variational approach with classical solutions (parabolic profile) of Stokes flow agree very well the second-order ELT derived by Tavakol et al.^30^ for a non-uniform channel. In this section, we suggest a more precise solution by substituting the velocity components and pressure derived by Hinojosa et al.^32^ into the formulation of Lagrangian and the ensuing Euler-Lagrange equation (in section Numerical simulations) for a constricting channel ($\lambda > 0$).

The velocity components and the pressure derived by Hinojosa et al.^32^ for non-uniform channel ($h_{+}(x)=h(x)$ and $h_{-}(x)=0$) are given by

39 $$\begin{aligned} u= & {} \frac{3}{2} \hat{u}(x) \left\{ 1- \left( 2\frac{y}{h(x)} -1 \right) ^2 \right\} \Bigg [ 1- \frac{\left( h(x)h^{\prime \prime }(x) -4 h^{'}(x)^2\right) }{40} \left\{ 1- 5 \left( 2\frac{y}{h(x)} -1 \right) ^2 \right\} \nonumber \\{} & {} + \frac{\left( h(x)h^{\prime \prime }(x) -6 h^{'}(x)^2 \right) }{6} \left( 2\frac{y}{h(x)} -1 \right) \Bigg ], \end{aligned}$$

40 $$\begin{aligned} v= & {} \frac{3}{2} \hat{u}(x) h^{'}(x)\left( \frac{y}{h(x)}\right) \left[ 1- \left( 2\frac{y}{h(x)} -1 \right) ^2 \right] , \end{aligned}$$

41 $$\begin{aligned} p= & {} g(x) + \frac{3}{2} \frac{\hat{u}(x) h^{'}(x)}{h(x)} \left[ \left\{ 1- 3\left( 2\frac{y}{h(x)} -1 \right) ^2 \right\} -2\left( 2\frac{y}{h(x)} -1 \right) \right] . \end{aligned}$$

Using similar formulations and same non-dimensional parameters mentioned in section Formulation of the problem and mathematical model we obtain the average pressure drop

42 $$\begin{aligned} \Delta \bar{P} = \int _{-1}^{1} \bar{P}(X) dX \end{aligned}$$

where the average pressure gradient is

43 $$\begin{aligned} \bar{P}(X)= & {} \frac{1}{2625 H^3} \Bigg [ -31500 +25200 {\delta }^2 (H^{\prime })^2 -35280 {\delta }^4 (H^{\prime })^4 -21564 {\delta }^6 (H^{\prime })^6 +16860 {\delta }^4 H (H^{\prime })^2 H^{\prime \prime } +5874 {\delta }^6 H (H^{\prime })^4 H^{\prime \prime } \nonumber \\{} & {} -960 {\delta }^4 H^2 (H^{\prime \prime })^2 +1268 {\delta }^6 H^2 (H^{\prime })^2 (H^{\prime \prime })^2 -310 {\delta }^6 H^3 (H^{\prime \prime })^3 +600 {\delta }^4 H^2 H^{\prime } H^{\prime \prime \prime } +1824 {\delta }^6 H^2 (H^{\prime })^3 H^{\prime \prime \prime } \nonumber \\{} & {} -296 {\delta }^6 H^2 H^{\prime } H^{\prime \prime } H^{\prime \prime \prime } -45 {\delta }^4 H^3 H^{\prime \prime \prime \prime } -162 {\delta }^6 H^3 (H^{\prime })^2 H^{\prime \prime \prime \prime } +28 {\delta }^6 H^4 H^{\prime \prime } H^{\prime \prime \prime \prime } \Bigg ] \end{aligned}$$

where $$H=H(X)$$.

**Figure 8:**
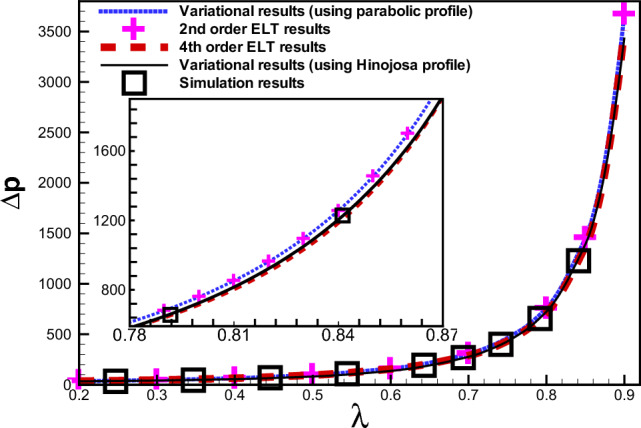
Comparison of the results obtained for $\delta =1$ for the dimensionless pressure drop using our variational approach based on the parabolic velocity profile (the dotted curve), our numerical finite-volume simulation (squares $\square$), and the second - ($+$) and fourth-order (the dashed line) ELT derived by Tavakol et al.^30^. The solid curve represents the results obtained using our variational approach based on the velocity profile derived by Hinojosa et al.^32^ for a constricting channel. The inset allows a better view of the five different sets of results in a smaller domain of $\lambda$.

Figure 8 presents the comparison of the variation of the dimensionless pressure drop with $\lambda$ as obtained using various-order ELT^30^, our variational approach with a parabolic profile derived in the section ’Formulation of the problem and mathematical model’, and our variational approach based on the velocity profile derived by Hinojosa et al.^32^. It is observed that for a constricting channel with $\delta =1$, the results for the dimensionless pressure drop derived from Eq. (42) agree very well with our numerical simulation results, and bounded by the second- order ELT which turns out to be identical to our results based on the variational approach using the parabolic velocity profile, and the fourth-order ELT results for $\lambda \in (0.78, 0.87)$. At the same time, the obtained results agree well with the fourth-order ELT and the numerical results in the range ($\lambda < 0.78) \bigcup (\lambda > 0.87$). Thus, for a channel with a sharp concavity (in the range $0.78 \le \lambda \le 0.87$) presented in the inset to Fig. 8 with $\delta =1$, the variational results based on the velocity profile derived by Hinijosa et al.^32^ significantly improve the estimate for the dimensionless pressure drop.

## Conclusions

Motivated by various applications in micro-fluidics, we consider a viscous low-Reynolds-number flow in a two-dimensional non-uniform channel. The aim of this study has been to determine the average pressure drop in constricting/expanding channel geometries using a variational approach and to compare the results with the ELT^30^ and finite-volume numerical simulations.

In this paper, we construct a Lagrangian for the Stokes flow in non-uniform channels in the form of an integral using the stationarity of a quantity composed of the work done by the pressure force and the entropy production^40^. Then, we impose the stationarity of the Lagrangian with respect to unknown functions to obtain a set of Euler-Lagrange equations. On further simplification, we achieve the average pressure drop as fully explicit functions of the wall geometry and the channel aspect ratio. The results derived here using the leading order variational approach agree very well with the second-order ELT derived by Tavakol et al.^30^, while the next order variational solution is in agreement with the fourth-order ELT. To validate the analytical results of our model, we perform 2-D finite-volume numerical simulations and find a good agreement between the theory and the simulations.

To summarize, we have proposed a variational approach in which the results obtained by the leading order are identical to the second-order lubrication approximation. This way we are able to obtain highly accurate results in an elegant manner. One more advantage of our variational approach over the ELT is that the pressure gradient can be determined from an explicit relationship using two simple ODEs. This approach is not restricted to $2-D$ asymmetric channels and can be utilised to obtain the average pressure drop between the two ends for channels with an arbitrary wall shape.

## Data Availability

The data set used and/or analyzed during the current study available from the corresponding author on reasonable request.
